# Spinal plasticity in stroke patients after botulinum neurotoxin A injection in ankle plantar flexors

**DOI:** 10.1002/phy2.173

**Published:** 2013-11-26

**Authors:** Claire Aymard, Louis-Solal Giboin, Alexandra Lackmy-Vallée, Véronique Marchand-Pauvert

**Affiliations:** 1 Service MPR, Centre Paris Sud, Fondation hospitalière Sainte MarieParis, France; 2 UPMC Univ Paris 06Er 6, F-75005, Paris, France

**Keywords:** Botulinum toxin, reciprocal inhibition, stroke

## Abstract

The effect of botulinum neurotoxin A (BoNT-A) in stroke patients' upper limbs has been attributed to its peripheral action only. However, BoNT-A depressed recurrent inhibition of lumbar motoneurons, likely due to its retrograde transportation along motor axons affecting synapses to Renshaw cells. Because Renshaw cells control group Ia interneurons mediating reciprocal inhibition between antagonists, we tested whether this inhibition, particularly affected after stroke, could recover after BoNT-A. The effect of posterior tibial nerve (PTN) stimulation on tibialis anterior (TA) electromyogram (EMG) was investigated in 13 stroke patients during treadmill walking before and 1 month after BoNT-A injection in ankle plantar flexors. Before BoNT-A, PTN stimuli enhanced TA EMG all during the swing phase. After BoNT-A, the PTN-induced *reciprocal facilitation* in TA motoneurons was depressed at the beginning of swing and reversed into inhibition in midswing, but at the end of swing, the *reciprocal facilitation* was enhanced. This suggests that BoNT-A induced spinal plasticity leading to the recovery of reciprocal inhibition likely due to the withdrawal of inhibitory control from Renshaw cells directly blocked by the toxin. At the end of swing, the enhanced *reciprocal facilitation* might be due to BoNT-induced modification of peripheral afferent inputs. Therefore, both central and peripheral actions of BoNT-A can modify muscle synergies during walking: (1) limiting ankle muscle co-contraction in the transition phase from stance to swing, to assist dorsiflexion, and (2) favoring it from swing to stance, which blocks the ankle joint and thus assists the balance during the single support phase on the paretic limb.

## Introduction

Post-stroke walking is characterized by coactivation of ankle flexors and extensors. Botulinum neurotoxin A (BoNT-A), indicated to paralyze overactive ankle plantar flexors, improves the temporal pattern of electromyographic (EMG) activity and the patients recover better alternated ankle muscle activities (Hesse et al. 1996). It is not known whether neurophysiological changes occur after muscular injection and the extent to which central phenomena participate in this functional improvement. Indeed, besides its well-known action at peripheral level, BoNT-A can affect central activity by influencing afferent inputs through its action on gamma motor endings (Filippi et al. 1993; Rosales et al. 1996), by inducing plastic changes following the blockade of the neuromuscular transmission (Abbruzzese and Berardelli 2006; Caleo et al. 2009), and finally through its retrograde transport along the motor axons (Antonucci et al. 2008; Torii et al. 2011). However, because their spinal excitability did not change after muscular injection in forearm muscles, it has been claimed that BoNT-A clinical effect is only limited to its peripheral action in stroke patients (Girlanda et al. 1997). However, it has been shown in arm muscles that BoNT-A reduces spastic co-contraction of non-injected antagonistic muscles (Gracies et al. 2009; Vinti et al. 2012). Moreover, we have recently shown in stroke patients that BoNT-A reduces recurrent inhibition of lumbar motoneurons (Marchand-Pauvert et al. 2013). These results raise again the question whether the clinical effects of BoNT-A in stroke patients are really limited to its peripheral action on overactive muscle or if it also induces spinal plasticity improving muscle synergies and functional recovery (Kaji 2013).

**Figure 1:**
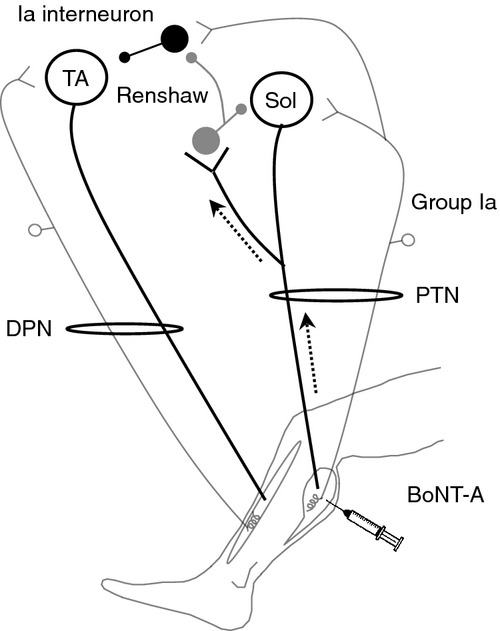
Schematic diagram of the spinal connections. Open circles represent spinal motoneurons innervating soleus (Sol) and tibialis anterior (TA). Dark and gray filled circles represent, respectively, group Ia interneurons mediating reciprocal inhibition from Sol to TA, and Renshaw cells activated by recurrent collaterals of Sol motoneurons, and which control group Ia interneurons inhibiting TA motoneurons. Gray line and open circle represent the muscle spindle group Ia afferents from Sol and TA, mediating H-reflex. TA sensory and motor axons run into the DPN, and those of Sol in PTN. BoNT-A was injected in triceps surae including Sol. Dotted line indicates the putative retrograde transport of BoNT-A. PTN, posterior tibial nerve; DPN, deep peroneal nerve; H-reflex, Hoffmann reflex; BoNT-A, botulinum neurotoxin A.

Stroke patients exhibit abnormal muscle synergies and co-activation of antagonistic muscles in particular. Accordingly, reciprocal inhibition between antagonists has been found strongly depressed after stroke (Yanagisawa et al. 1976; Crone et al. 2003). It has been shown in healthy subjects that reciprocal inhibition is modulated during the gait cycle and may *help to inactivate antagonistic motoneurones in the appropriate phases of the walking cycle. Depression of the inhibition in the opposite phases may help to ensure an unhindered activation of the motoneurones by descending and segmental excitatory inputs* (Petersen et al. 1999). Because reciprocal inhibition is depressed after stroke (Yanagisawa et al. 1976; Crone et al. 2003) and the ankle muscles are co-activated during post-stroke walking, one would expect abnormal modulation of reciprocal inhibition between ankle muscles during post-stroke walking. Moreover, triceps surae being mostly injected in stroke patients, we investigated reciprocal inhibition in ankle dorsiflexors. First, because we wanted to further investigate how changes in afferent inputs from an injected muscle influences the activity of motoneurons supplying a non-injected muscle. Second, because Ia inhibitory interneurons mediating reciprocal inhibition between ankle muscles are controlled by Renshaw cells (Baret et al. 2003; Fig. 1), we wondered whether BoNT-A may help to reactivate reciprocal group Ia interneurons by blocking Renshaw cells (Marchand-Pauvert et al. 2013). We therefore tested reciprocal inhibition from ankle extensors to flexors during post-stroke walking, before and after BoNT-A muscular injection in ankle extensors.

The so-called *reciprocal inhibition* between wrist muscles was found unchanged after toxin injection in stroke patients (Girlanda et al. 1997). However, the inhibition of motoneurons supplying the injected muscle was investigated, which makes it difficult to interpret the EMG recordings due to the plastic changes at muscular and motoneuron levels (Abbruzzese and Berardelli 2006; Caleo et al. 2009). On the other hand, spastic co-contraction of non-injected antagonistic muscle was reduced at elbow level after BoNT-A, possibly due to plastic changes at spinal level (Gracies et al. 2009; Vinti et al. 2012). Therefore, it seems that the effects of BoNT-A may be different depending on the target joint. We thus further addressed the question whether BoNT-A influences activity of non-injected antagonistic muscle and its repercussions on spinal excitability in stroke patients, who were tested while walking, to explore the effects of BoNT-A in a functional context.

## Methods

### Ethical approval

The study conformed to the standards set by the latest revision of the Declaration of Helsinki and has been approved by the ethics committee of Pitié-Salpêtrière Hospital (CPP-Ile-de-France VI). Seventeen stroke patients (five females) were included in the protocol, all of whom had given informed written consent to the experimental procedures.

### Patients

Inclusion criteria were spastic leg paresis, marked increase in tone at ankle level, a minimum 1-month interval since stroke, and clinical prescription for BoNT-A injection only in ankle plantar flexors. Exclusion criteria included BoNT-A injection within the previous 4 months and in other muscles than ankle plantar flexors, previous alcohol or phenol blocks, surgical intervention, or casting of the lower limb: fixed contractures in the limb(s) or profound atrophy of muscle(s) to be injected. We had to exclude 4/17 patients because soleus reflex response was so large that it contaminated tibialis anterior (TA) EMG activity, which was no longer interpretable due to concomitant compound potential with similar shape in TA and soleus EMG (cross talk; Hutton et al. 1988). The experimental protocol was thus possible in only 13 patients (mean age: 52.8 ± 3.0 years old; range: 25–64), and mean interval since stroke was 29.3 ± 14.8 months (range: 1–180). Ongoing treatments (physical therapy and medication) remained unchanged. Each patient was assessed 2 times: before (preBoNT-A) and 1 month after BoNT-A injection (postBoNT-A), i.e.*,* when clinical effects of BoNT-A injection are seen in most patients (Kaji et al. 2010). BoNT-A was injected by C. A. (among the authors) into triceps surae in all patients, and into tibialis posterior in six patients, according to the clinical prescription. The injection site was guided by EMG to localize motor end plates and muscle hyperactivity. Doses were established according to the guidelines of the Worldwide Education and Awareness for Movement Disorders (WEMOVE, http://www.mdvu.org). Although the doses may be considered low, muscle tone was reduced by two points in 12/13 patients, and by one point in the remaining patient (Table 1).

**Table 1:** Clinical features of the patients

		Lesion			BONT-A					Spasticity		Inhibition	
					
Patients	Speed	Time	Type	Site	Type	Soleus	MG	LG	TP	Pre	Post	Pre	Post
1.F.62	0.6	5	Isch.	L	ABO	3/300	1/100	1/100	1/50	3	1	90.6	82.0
2.M.53	2.0	2	Isch.	L	ABO	3/300	1/100	1/100	–	2	0	99.2	76.8
3.M.64	0.6	3	Isch.	R	ABO	3/300	1/150	1/150	1/150	2	0	69.5	86.5
4.M.42	2.6	1	Hem.	R	ABO	3/300	1/100	1/100	–	2	0	83.2	80.0
5.F.53	1.0	5	Isch.	R	ABO	3/300	1/100	1/100	–	3	1	103.0	50.1
6.M.50	0.6	180	Isch.	R	ABO	3/300	1/100	1/100	1/150	3	1	74.9	40.4
7.M.63	1.8	7	Isch.	R	ABO	3/300	1/100	1/100	–	3	1	83.6	86.1
8.M.49	0.8	3.5	Isch.	L	ABO	3/300	1/100	1/100	1/150	2	0	101.8	80.4
9.M.61	0.8	16	Hem.	L	ABO	3/300	1/150	1/150	1/150	3	1	176.8	94.0
10.M.62	1.4	11	Isch.	L	ABO	3/300	1/100	1/100	–	3	1	114.6	77.7
11.M.25	1.6	108	Hem.	L	ONA	3/60	1/20	1/20	–	2	0	60.1	63.4
12.M.55	2.0	24	Hem.	L	ABO	3/300	1/100	1/100	–	3	2	72.7	75.3
13.M.47	1.0	15	Hem.	L	ABO	3/200	1/100	1/100	1/100	2	0	98.7	73.4

*Patients*: rank, gender (M, male; F, female), and age of the patients at the time of the investigation (years); *Speed*: walking speed before and after BoNT-A; *Lesion*: Time = Time lapse between stroke and the first electrophysiological investigation, before BoNT-A (months); *Type* = origin of the lesion (Isch., ischemia; Hem., hemorrhage); *Site* = cerebral hemisphere affected; *BoNT-A*: type indicates the type of toxin injected (ABO: abobotulinumtoxinA; ONA: onabotulinumtoxinA); number of injection sites/dose (UI) in muscles receiving BoNT-A: soleus, MG (medial gasctrocnemius), LG (lateral gasctrocnemius), and TP (tibialis posterior); *Spasticity*: estimation of muscle tone (Ashworth score) in soleus before (Pre) and 1 month after toxin injection (Post); *Inhibition*: maximal inhibition/less facilitation observed before (Pre) and 1 month after toxin injection (Post), whatever the walking phase.

### Recordings

EMG was recorded with bipolar surface electrodes (DE-2.1; Delsys Inc., Natick, MA) placed over the muscle bellies of soleus (medial part of the posterior aspect of the leg, 2–3 cm below the gastrocnemius muscles) and TA (medial part of the anterior aspect of the leg, 10–15 cm below the patella). EMG activity was amplified (10,000×; Delsys Bagnoli System; Delsys Inc.), and filtered (bandwidth 20–450 Hz), before being digitally stored (2-kHz sampling rate) on a personal computer for off-line analysis (Power 1401; CED, Cambridge, UK). Recordings were undertaken during treadmill locomotion (Biodex Medical Systems Inc., Shirley, NY). Because of foot drop on paretic side and slow speed, the patients contacted the ground with the forefoot. The pressure transducer, detecting the ground contact, was thus placed at the middle and external aspect of the sole to time the beginning of stance phase. The patients first walked on the treadmill for 5–10 min before recordings, to accustom themselves to treadmill walking and to determine their comfortable speed (mean walking speed 1.3 ± 0.2 km h^−1^, range 0.6–2.0; Table 1). This speed was not their maximum possible speed, but they felt secure and were able to walk for 2–3 min (∼recording duration) without fatigue. They were asked to walk at the same speed after BoNT-A. All patients were able to walk freely and investigations were performed without bodyweight support.

### Stimulation

One-millisecond rectangular electrical pulses were delivered through surface electrodes by constant current stimulators (DS7A; Digitimer Ltd, Hertfordshire, UK). The current crossed posterior tibial nerve (PTN) through a 7-cm^2^ brass hemispheric electrode placed in the popliteal fossa (cathode) and a 21-cm^2^ brass plaque above the patella. The deep peroneal nerve (DPN) was stimulated using two 7-cm^2^ brass hemispheres: one placed behind the head of the fibula (cathode) and the second one, on the anterior aspect of the leg, 5–7 cm below the patella. The optimal stimulation sites were determined clinically by tendon palpation of soleus and TA. PTN stimulation intensity was adjusted according to the threshold intensity for direct motor response in soleus EMG activity (xMT, motor threshold): stimulus intensity was 1 xMT in all patients, and stimuli at 1.5 xMT were also tested in four patients. DPN stimulation was adjusted to evoke a sizeable Hoffmann reflex (H-reflex) in TA EMG and to compare its latency to that of soleus H-reflex (see below).

## Experimental Procedures

**Figure 2:**
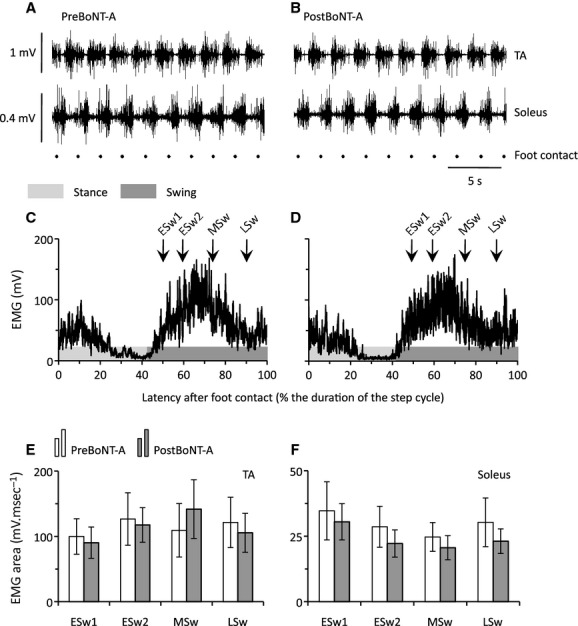
EMG activities during post-stroke walking. (A, B) EMG in tibialis anterior (TA), soleus, and signal for foot contact on the paretic side of one patient walking at 1 km h^−1^, recorded simultaneously before (A) and 1 month after BoNT-A (B). (C, D) Rectified and averaged (10 steps) TA EMG in the same patient before (C) and after BoNT-A (D) plotted against normalized latency after foot contact. Vertical arrows indicate when stimuli were delivered in early (ESw1–2), mid- (MSw) and late swing (LSw). Light and dark gray areas indicate, respectively, stance and swing phase. (EF) Mean background EMG (±1 SEM) in TA (E) and soleus (F) before (open columns) and after BoNT-A (gray columns) in the group of 13 patients. EMG, electromyogram; BoNT-A, botulinum neurotoxin A.

The effect of PTN stimulation on TA EMG was tested during treadmill walking. Stimuli were triggered by the signal from the pressure transducer, at four delays after foot contact during the swing phase when TA was activated: at the onset of TA activity (Early swing 1, ESw1), at maximal TA activity in early swing (Early swing 2, ESw2), in mid-swing (MSw) and late swing (LSw; Fig. 2). These delays were determined according to the walking pattern and did not significantly differ between the two experiments: (1) mean delay for ESw1 was 1037 ± 100 msec (600–1720 msec) before BoNT-A versus 992 ± 104 msec (600–1800 msec) after BoNT-A (*P* = 0.47), (2) for ESw2, 1167 ± 110 msec (650–1820 msec) *versus* 1115 ± 116 msec (640–2000 msec; *P* = 0.44), (3) for MSw, 1431 ± 122 msec (870–2200 msec) *versus* 1393 ± 128 msec (710–2500 msec; *P* = 0.61), and (4) for LSw, 1649 ± 125 msec (1090–2700) *versus* 1651 ± 150 ms (1020–2800 msec; *P* = 0.98). The speed and the average step cycle time (1888 ± 164 msec *versus* 1912 ± 193 msec; *P* = 0.87) were similar before and after BoNT-A, and PTN stimuli were thus delivered on average at 53 ± 1 (ESw1), 60 ± 2 (ESw2), 75 ± 2 (MSw), and 91 ± 2 (LSw)% the duration of the gait cycle during the two experiments. One recording session consisted in delivering 20 PTN stimuli at one delay after ground contact. Two recording sessions were performed at each delay: two sessions for 1 delay × 4 delays = eight recording sessions in each patient. Recordings of control (without PTN stimulation) and conditioned EMG (with PTN stimulation) were randomly alternated during each session at a frequency determined by foot contact (0.3–0.5 Hz).

### EMG analysis

**Figure 3:**
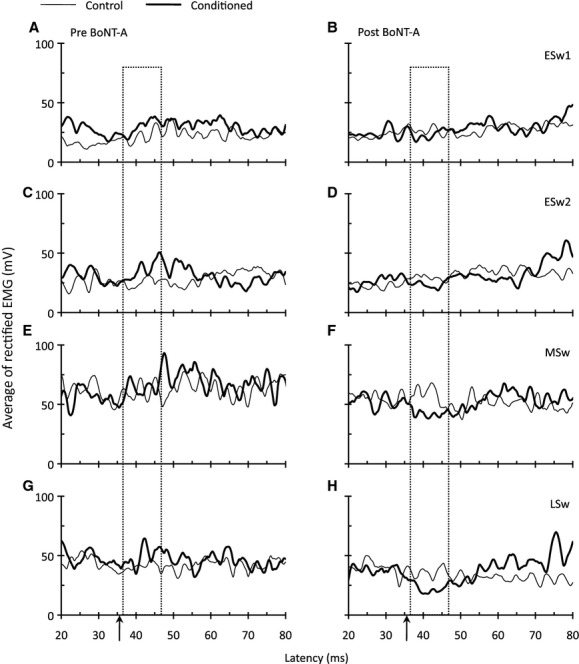
PTN-induced EMG modulation of TA EMG before and after BoNT-A. Rectified and averaged (*N* = 40) TA EMG without (control) and after PTN stimuli (conditioned) at 1 xMT delivered in early swing ESw1–2 (A, B, C, D) midswing (MSw) (E, F) and late swing (LSw) (G, H), plotted against the latency after stimulation, in one patient before (A, C, E, G) and after BoNTA (B, D, F, H). Vertical arrows indicate the H-reflex latency and dashed lines, the window of analysis. PTN, posterior tibial nerve; TA, tibialis anterior; EMG, electromyogram.

TA EMG activity was rectified and averaged: *N* = 40 traces of control EMG and 40 of conditioned EMG for each delay investigated. Usually, the analysis is calibrated according to the arrival time of muscle spindle group Ia afferent fibers at motoneuron level. For this study, the calculation should have been based on the latency of TA H-reflex, the distance between DPN and PTN stimulation sites, and the conduction velocity of their group Ia fibers. The mean latencies of TA and soleus H-reflexes were similar (32.5 ± 0.8 and 31.6 ± 1.1 msec, respectively; *P* = 0.43). However, it was possible to evoke an H-reflex in TA only in 9/13 patients, and in soleus in all of them. Hence, for interindividual comparisons, PTN-induced modulation of TA EMG was analyzed within the window of analysis determined according to patient soleus H-reflex latency. The central latency for reciprocal inhibition is about 1 msec (Crone et al. 1987), and its duration is about 10 msec (Pierrot-Deseilligny et al. 1981; Capaday et al. 1990; Petersen et al. 1999). Therefore, the window of analysis started from the latency of soleus H-reflex + 1 msec and lasted 10 msec in all patients (vertical dashed lines, Fig. 3). Both control and conditioned EMG were evaluated over the same window of analysis, and the area of conditioning EMG was normalized to the mean area of control EMG recorded during the same session. The ratio gave a quantitative estimation of PTN-induced modification of TA motoneuron excitability (ratio > 100% indicates motoneuron facilitation and <100%, motoneuron inhibition).

### Statistical analysis

Two-tailed paired *t-*tests were performed to compare control and conditioned EMG in each individual, locomotor parameters before and after BoNT-A and the H-reflex latencies in the group. Because normality and homogeneity of variances were not respected in the group data, nonparametric Friedman tests were performed to compare pre- and post-BoNT-A recordings, background EMG activity, conditioned TA and soleus EMG and intensity of PTN stimulation. If the tests provided significant *P* values, Wilcoxon signed rank tests were performed for comparison of two means. Spearman's rank correlation coefficient was calculated to test the influence of the time since stroke and the walking speed on reciprocal inhibition and its changes after BoNT-A. The levels of *reciprocal facilitation* and inhibition in the group, in each condition (walking phase, preBoNT-A, postBoNT-A), were individually tested using one-sample *t*-tests. Tests were performed using StatEL software (http://www.adscience.eu) and the significance level was set at *P* < 0.05. The mean data are indicated ± 1 standard error of the mean (SEM).

## Results

### Walking patterns

EMG temporal pattern (alternating TA and soleus activity) was more normal after BoNT-A, with a reduction in soleus premature activity: before BoNT-A, soleus activity started ∼500 msec before the foot contact and, after BoNT-A, it started ∼300 msec before the foot contact in the patient illustrated in Figure 2A and B. Moreover, the silent period between 2 TA EMG bursts was sharper after BoNT-A (Fig. 2A–D, same patient): the short silent period around 40% the duration of the gait cycle before BoNT-A (Fig. 2C) which was more pronounced between 25 and 40% after BoNT-A (Fig. 2D). Better timing with less premature activity in soleus and/or silent period in TA after BoNT-A, i.e*.,* less co-contraction between antagonists, was observed in 9/13 patients, as much as observed previously (Hesse et al. 1996). In three patients, we observed no change: in two patients, the temporal pattern was normal before BoNT-A, and in the third one, the poor synchrony did not improve after BoNT-A. In the remaining patient (9.M.61 in Table 1), there was no detectable surface EMG activity in soleus after BoNT-A.

On average (13 patients), the level of TA and soleus EMG did not change between pre- and post-BoNT-A recordings (Fig. 2E and F; *P* = 0.28 and 0.42, respectively). In a previous study, EMG was greater after BoNT-A (Hesse et al. 1996) but this was probably due to an increase in walking speed. Most of our patients could walk faster after BoNT-A, but the speed was similar to compare spinal excitability under the same conditions.

### PTN-induced modifications in TA EMG

In one patient (Fig. 3A and B), PTN stimulation did not modify TA EMG activity before and after BoNT-A during ESw1 (*P* = 0.20 and 0.85). However, the mean level of conditioned EMG was above the control before BoNT-A and below after. Similar results were obtained in ESw2 (Fig. 3C and D), MSw (Fig. 3E and F) and LSw (Fig. 3G and H): PTN-induced TA EMG facilitation before BoNT-A was statistically significant only in ESw2 (129.8 ± 14.5%, *P* < 0.05; Fig. 3C) and EMG suppression after BoNT-A was statistically significant only in MSw (73.4 ± 4.6%, *P* < 0.01; Fig. 3H).

**Figure 4:**
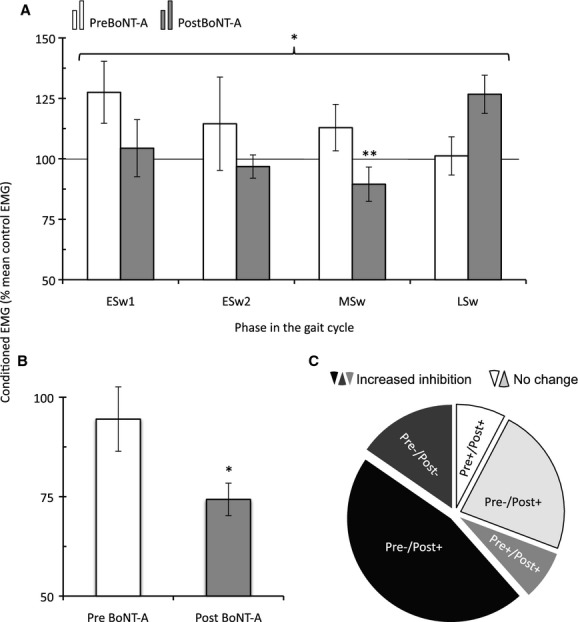
Reverse effects of PTN stimulation after BoNT-A. (A) Mean area (% the mean control EMG) of TA EMG after PTN stimuli (1 xMT) delivered in early (ESw1–2), mid- (MSw) and late swing (LSw), before (open columns) and after BoNT-A in the group of 13 patients. (B) Maximal inhibition (mean surface <100%) in the group, whatever the gait phase. Error bars are ±1 SEM. *P < 0.05. (C) Pie chart indicating the number of patients exhibiting no change (white and light gray portions) or increased PTN-induced inhibition (black and dark gray portions) and good locomotor synchrony before and after BoNT-A (Pre+/Post+) or bad locomotor synchrony before (Pre-/Post+) or no improvement of synchrony (Pre-/Post-). PTN, posterior tibial nerve; TA, tibialis anterior; EMG, electromyography.

Nonparametric Friedman test revealed that the PTN-induced TA EMG facilitation was significantly reduced after BoNT-A (13 patients; *P* < 0.05; Fig. 4), particularly in MSw when it was reversed into inhibition (112.9 ± 9.6 vs. 89.5 ± 7.1%; *P* < 0.01). TA EMG facilitation was statistically significant only in ESw1 before BoNT-A (127.5 ± 12.8; *P* < 0.05) and in LSw after BoNT-A (126.7 ± 7.9%, *P* < 0.01).

Before BoNT-A, PTN stimulation facilitated TA EMG activity in 9/13 patients and depressed it in 7/13, in different walking phases. We therefore compared the maximal level of inhibition in TA before and after BoNT-A in each patient, regardless the phase investigated, or when the facilitation was less in the six patients who did not exhibit any inhibition before BoNT-A. First, we did not find any correlation between the walking speed and the maximal level of inhibition (Spearman's rank correlation coefficient *r* = −0.08, *P* = 0.77) neither with it changes after Bo-NT-A (*r* = −0.21, *P* = 0.46). Second, we found the TA EMG suppression significantly greater after BoNT-A (Fig. 4B: 94.5 ± 8.1 vs. 74.3 ± 4.1%; *P* < 0.05; 9/13 patients). While we could not perform a distribution test because of the small size of patient sub-groups, we found almost half patients (6/13) exhibiting better alternating TA/soleus activities combined with stronger inhibition after BoNT-A (Pre-/Post+ group, Fig. 4C).

### Relation between maximal inhibition in TA and muscle tone in soleus

We tested whether the maximal PTN-induced TA inhibition was related to muscle tone in soleus. No significant relation was found between the level of inhibition and muscle tone both before and after BoNT-A (*P* = 0.32 and 0.61, respectively). Moreover, almost all patients exhibited −2 points after BoNT-A, according to the Ashworth scale, whatever the change in TA inhibition. Lastly, we did not find any influence of time since stroke onto the changes in reciprocal inhibition after BoNT-A (Spearman's rank correlation coefficient *r* = 0.15, *P* = 0.61).

### Monitoring of conditioning stimuli

**Figure 5:**
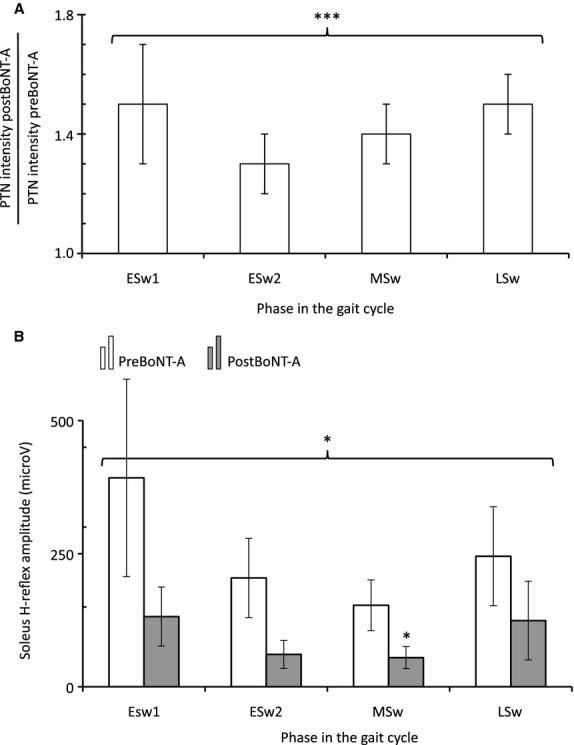
PTN stimulation intensity and soleus H-reflex. (A) Ratio between the intensities of PTN stimuli after and before BoNT-A delivered in early (ESw1–2), mid- (MSw) and late swing (LSw). (B) Amplitude of soleus H-reflex before (open columns) and after BoNT-A (gray columns) in the different phases of the gait cycle. Error bars are ±1 SEM. *P < 0.05, ***P < 0.001. PTN, posterior tibial nerve. ESw1, Early swing 1; PTN, posterior tibial nerve.

The intensity of PTN stimulation was stronger after BoNT-A (*P* < 0.001; Fig. 5A). On average, the threshold for motor response in soleus after BoNT-A was 1.4 ± 0.1 time more than that before BoNT-A (range 0.9–2.2). Stimuli at 1.0 and 1.5 xMT were tested before BoNT-A in four patients in ESw2 and MSw, i.e.*,* when PTN-induced facilitation was less or even reversed into inhibition. In one patient, the resulting soleus H-reflex was so increased at 1.5 xMT that it was not possible to distinguish between modulation of TA EMG and cross talk from soleus. In the other three patients, PTN stimuli at 1 xMT did not modulate TA EMG significantly (100.1 ± 8.6 and 95.1 ± 11.5% in ESw2 and MSw, respectively). Increasing stimulus intensity to 1.5 xMT produced no change (one patient) or TA EMG facilitation (two patients; on average 132.1 ± 18.3 and 126.0 ± 23.0% in ESw2 and MSw, respectively).

Friedman test revealed a significant decrease in soleus H-reflex after BoNT-A (*P* < 0.05), especially in MSw (*P* < 0.05; Fig. 5B).

## Discussion

This study has first shown that PTN stimulation increases TA EMG during the swing phase in stroke patients. The facilitation was evaluated within a window of analysis when PTN stimulation normally depresses TA EMG in healthy subjects. This inhibition is produced by group Ia interneurons mediating reciprocal inhibition (Petersen et al. 1999). Reciprocal inhibition of soleus motoneurons has been found depressed in stroke patients at rest, and even reversed into excitation (Yanagisawa et al. 1976; Crone et al. 2003), as we observed here during walking before BoNT-A. These changes were closely related to motor abilities, including walking: the better the functional recovery, the greater the reciprocal inhibition, but not to spasticity (Okuma and Lee 1996; Bhagchandani and Schindler-Ivens 2012), as we found here (the level of reciprocal inhibition recovery was not correlated to spasticity but probably to functional recovery).

### *Reciprocal facilitation* during post-stroke walking

Reciprocal inhibition of TA motoneurons has been explored to a lesser extent than soleus for methodological reasons (difficulty to evoke TA H-reflex). One study on three patients reported no change (Yanagisawa et al. 1976). Our method allowed us to investigate a larger group of patients. We could demonstrate reciprocal inhibition of TA motoneurons in half the patients before BoNT-A, which further supports the idea that reciprocal inhibition might be less affected for TA than for soleus. However, on average, the result was much the same as for soleus (Crone et al. 2003), i.e*.,* reciprocal inhibition of TA motoneurons is depressed and may even be reversed into excitation after stroke. In healthy subjects, *reciprocal excitation* of TA motoneurons has been attributed to concomitant reflex response in soleus (cross talk; Hutton et al. 1988). However, we have excluded the patients in whom TA response was due to cross talk. The neurophysiological mechanism underlying this *reciprocal facilitation* after stroke is still unclear. It may result from disrupted supraspinal control (Crone et al. 2003). Alternatively, *reciprocal facilitation* during post-stroke walking could be mediated by group Ib spinal pathways. Group Ib inhibition is reversed into excitation after stroke (Delwaide and Oliver 1988), and group Ib excitatory pathway is specifically activated during walking (Stephens and Yang 1996; Marchand-Pauvert and Nielsen 2002). This might explain why reciprocal inhibition of TA motoneurons was found unchanged in stroke patients at rest (Yanagisawa et al. 1976), and reversed into excitation during walking in this study.

### Recovery of reciprocal inhibition after BoNT-A

Our second and main result is that *reciprocal facilitation* was depressed in ESw and even reversed into inhibition in MSw after BoNT-A. Technical reasons could account for this modification: (1) Background EMG influences reciprocal inhibition (Petersen et al. 1999) but it did not change between recordings. (2) Because BoNT-A blocks part of neuromuscular junctions, the motor response is less (On et al. 1999) and its threshold intensity can be higher. Accordingly, PTN stimulation intensity was stronger after BoNT-A. However, similar stimuli before BoNT-A resulted in an increase in *reciprocal facilitation* (Yanagisawa et al. 1976), not its reversal. Therefore, spinal plasticity after BoNT-A likely accounts for the recovery of reciprocal inhibition. Indeed, the excitability of the central nervous system is modified after BoNT-A (Currà et al. 2004; Rosales and Dressler 2010). Because extra- and intrafusal fibers are similarly affected, BoNT-A central effects were mainly attributed to the resulting change in muscle spindle afferent inputs (Rosales et al. 1996). Accordingly, soleus H-reflex was depressed after BoNT-A, possibly due to reduced spindle discharge (Manni et al. 1989; On et al. 1999). H-reflex decrease was not paralleled by significant depression of EMG activity. This suggests possible specific changes in the excitability of synapses between group Ia afferents and motoneurons, involving inhibitory nonreciprocal group Ib interneurons and post activation depression because both can limit H-reflex (Pierrot-Deseilligny and Burke 2012); increased presynaptic inhibition of group Ia afferents mediating H-reflex, is unlikely because it did not recover after BoNT-A in stroke patients (Girlanda et al. 1997; Panizza et al. 2000).

### Possible neurophysiological mechanism

*Reciprocal inhibition* in forearm flexors recovered after BoNT-A in patients with dystonia or essential tremor (Priori et al. 1995; Modugno et al. 1998), but not in stroke patients (Girlanda et al. 1997). Precisely, only the late phase, reflecting group Ia presynaptic inhibition, changed but not the early phase corresponding to postsynaptic *reciprocal inhibition* (Pierrot-Deseilligny and Burke 2012). In stroke patients, the clinical effects of BoNT-A was thus attributed to its peripheral action because the spinal excitability did not change (Girlanda et al. 1997). However, the results in lower limbs do not support this hypothesis. (1) Recurrent inhibition mediated by soleus-coupled Renshaw cells decreased after BoNT-A in soleus (Marchand-Pauvert et al. 2013), much as in animal preparations (Hagenah et al. 1977; Wiegand and Wellhöner 1977). (2) Here, we reported that *reciprocal facilitation* is depressed and reciprocal inhibition of TA motoneurons recovers. The discrepancy between the results in upper and lower limbs can be explained by different spinal organization. *Reciprocal inhibition* between wrist flexors and extensors is not reciprocal in origin, but mediated by nonreciprocal group I interneurons, while inhibition between TA and soleus is mediated by reciprocal group Ia interneurons. These two groups of interneurons differ by their mediator, their afferent inputs, and by the fact that Renshaw cells project onto reciprocal group Ia interneurons only (Aymard et al. 1995; Floeter et al. 1996; Crone et al. 2001; Wargon et al. 2006). Indeed, reciprocal inhibition of TA motoneurons is deeply suppressed by activation of soleus-coupled Renshaw cells (Baret et al. 2003; Fig. 1).

Less recurrent inhibition could lead to more inhibition by reciprocal group Ia interneurons. Accordingly, spastic co-contraction of non-injected antagonistic muscle is reduced after BoNT-A at elbow level in stroke patients. This has been attributed to possible reinforcement of reciprocal inhibition through a decreased recurrent inhibition from the injected muscles (Gracies et al. 2009; Vinti et al. 2012). BoNT-A did not modify the excitability of reciprocal group Ia interneurons in an animal preparation, but the excitability of Renshaw cells was also not modified (Hagenah et al. 1977). Therefore, it seems that Renshaw cells need to be blocked by the toxin for an effect on reciprocal inhibition of antagonistic motoneurons. The changes in peripheral inputs after BoNT-A are insufficient to influence disynaptic inhibition, especially in stroke patients (Girlanda et al. 1997), and it is likely that the recovery of reciprocal inhibition of TA motoneurons during post-stroke walking is due to depression of recurrent inhibition by BoNT-A. This further supports BoNT-A direct central action on spinal circuitry, involving its retrograde transport along motor axons, which allows the blockade of Renshaw cells (Mazzocchio et al. 2007; Marchand-Pauvert et al. 2013; Kaji 2013; Fig. 1).

### Enhanced *reciprocal facilitation* in LSw and mechanisms

Our third and last finding is the enhanced *reciprocal facilitation* in LSw after BoNT-A. Group Ib excitation seems to be particularly enhanced after stroke, not only because of depressed reciprocal inhibition but also because of disrupted corticospinal inhibitory control of excitatory group Ib interneurons (Pierrot-Deseilligny and Burke 2012). However, this does not explain why *reciprocal facilitation* was particularly strong in LSw after BoNT-A. The chosen delay was very close to the foot contact, i.e.*,* the beginning of stance. At this delay, ankle extensors are beginning to be activated, especially in stroke patients in whom these muscles are prematurely contracted (Perry et al. 1978; Knutsson and Richards 1979). In healthy subjects at rest, gastrocnemius medialis group Ib afferents produce excitation in TA motoneurons (Pierrot-Deseilligny et al. 1981). Given that group Ib excitatory pathways are especially open during walking (Stephens and Yang 1996; Marchand-Pauvert and Nielsen 2002), and likely enhanced after stroke (Delwaide and Oliver 1988; Crone et al. 2003; Pierrot-Deseilligny and Burke 2012), *reciprocal facilitation* during LSw might not be surprising due to the co-contraction of ankle flexors and extensors. This excitation has not been reported during normal walking (Petersen et al. 1999) and, not observed during post-stroke walking before BoNT-A. Therefore, toxin injection might have induced other plastic changes, which particularly manifested during LSw. Plasticity involving Renshaw cells at this level is unlikely because they control motoneurons and reciprocal group Ia interneurons only, not group Ib interneurons (Pierrot-Deseilligny and Burke 2012). All other things held constant, a mechanism involving Renshaw cells and/or changes in motoneuron excitability would have led to similar modification all during the swing phase. Because of its peripheral action, BoNT-A might interfere with the normal relationship of group Ib activation by contraction. Given the extreme sensitivity of tendon organs (Binder et al. 1977), it is quite likely that contraction force and EMG are disproportionally affected such that there is relatively greater group Ib activation for force. Alternatively, axon growth in tendon organs, reported in neonatal mouse after BoNT-A, leads to greater group Ib activity (Hopkins 1984). If similar sprouting occurred in stroke patients, one would expect an increase in group Ib afferent inputs. Last possibility, group Ib interneurons receive peripheral inputs of various origin (group Ia, Ib, cutaneous, articular etc.) and modification of any peripheral input after BoNT-A would enhance *reciprocal facilitation*.

### Implication in functional recovery

The recovery of reciprocal inhibition was accompanied by a more normal temporal pattern of activity during walking. Indeed, the depression of *reciprocal facilitation* and the recovery of reciprocal inhibition of TA particularly manifested in ESw and MSw might contribute to a better timing of ankle dorsiflexor activity and transition from stance to swing phase. Therefore, the improved muscle synchrony during walking cannot be attributed to toxin peripheral action on ankle plantar flexors only. Accordingly, our results suggest that the toxin also influences spinal excitability, facilitating a more normal activity in ESw and MSw (Petersen et al. 1999). On the other hand, *reciprocal facilitation* in LSw after BoNT-A reinforces the co-contraction between antagonists, and possibly counteracts the transition phase from swing to stance. Contrary to ESw, the co-contraction between antagonists might be interesting in LSw to block the ankle position when the bodyweight shifts onto the paretic leg, to ensure the upright posture. Accordingly, co-contraction of ankle antagonists occurs when the balance is perturbed during normal walking, especially in early stance (Misiaszek et al. 2000; Iles et al. 2007). During such a co-contraction, reciprocal inhibition is depressed (Nielsen and Kagamihara 1992). The risk of falling is particularly increased after stroke (Weerdesteyn et al. 2008), and co-contraction of antagonists when the upright posture is particularly challenged at the beginning of the single limb support on the paretic side could minimize this. The role of co-contractions in movement disorders after stroke is still debated (Knutsson and Richards 1979; Knutsson and Mårtensson 1980; Hidler et al. 2007). Muscle weakness is the most limiting parameter for motor recovery, but is not explained by excessive antagonist activity (Newham and Hsiao 2001; Neckel et al. 2006). Therefore, *reciprocal facilitation* after BoNT-A in LSw favors co-contraction at ankle level, and this might contribute to the maintenance of upright posture.

## Conclusions

BoNT-A muscular injection improves muscle synchrony during post-stroke walking, not only by paralyzing partially spastic muscles but also by influencing spinal excitability through direct and indirect central actions: (1) blockade of the synapse between motor axons and Renshaw cells (Marchand-Pauvert et al. 2013), which facilitates reciprocal inhibition recovery, and (2) enhanced group Ib excitations likely due to toxin-induced changes in peripheral inputs (Currà et al. 2004; Rosales and Dressler 2010). The resulting changes in spinal excitability influences muscle synergies by (1) limiting co-contraction between antagonistic muscles when necessary during the transition phase from stance to swing, and (2) facilitating the same co-contraction during the transition phase from swing to stance to block the ankle joint and assist the maintenance of upright posture during the supporting phase on the paretic side. These effects are mainly due to the spinal circuitry organization, which allows plasticity and makes possible the activation of relevant neural networks for the functional recovery. BoNTA muscular injection does not partially paralyze spastic muscles only but also promotes profitable post-lesional plasticity. Our results show the importance of taking into account the spinal circuitry behind the injected muscle because it may condition the plasticity after BoNT-A injection and its clinical effects.

## References

[b1] Abbruzzese G, Berardelli A (2006). Neurophysiological effects of botulinum toxin type A. Neurotox. Res.

[b2] Antonucci F, Rossi C, Gianfranceschi L, Rossetto O, Caleo M (2008). Long-distance retrograde effects of botulinum neurotoxin A. J. Neurosci.

[b3] Aymard C, Chia L, Katz R, Lafitte C, Pénicaud A (1995). Reciprocal inhibition between wrist flexors and extensors in man: a new set of interneurones?. J. Physiol.

[b4] Baret M, Katz R, Lamy JC, Pénicaud A, Wargon I (2003). Evidence for recurrent inhibition of reciprocal inhibition from soleus to tibialis anterior in man. Exp. Brain Res.

[b5] Bhagchandani N, Schindler-Ivens S (2012). Reciprocal inhibition post-stroke is related to reflex excitability and movement ability. Clin. Neurophysiol.

[b6] Binder MD, Kroin JS, Moore GP, Stuart DG (1977). The response of Golgi tendon organs to single motor unit contractions. J. Physiol.

[b7] Caleo M, Antonucci F, Restani L, Mazzocchio R (2009). A reappraisal of the central effects of botulinum neurotoxin type A: by what mechanism?. J. Neurochem.

[b8] Capaday C, Cody FW, Stein RB (1990). Reciprocal inhibition of soleus motor output in humans during walking and voluntary tonic activity. J. Neurophysiol.

[b9] Crone C, Hultborn H, Jespersen B, Nielsen J (1987). Reciprocal Ia inhibition between ankle flexors and extensors in man. J. Physiol.

[b10] Crone C, Nielsen J, Petersen N, Tijssen MA, van Dijk JG (2001). Patients with the major and minor form of hyperekplexia differ with regards to disynaptic reciprocal inhibition between ankle flexor and extensor muscles. Exp. Brain Res.

[b11] Crone C, Johnsen LL, Biering-Sørensen F, Nielsen JB (2003). Appearance of reciprocal facilitation of ankle extensors from ankle flexors in patients with stroke or spinal cord injury. Brain.

[b12] Currà A, Trompetto C, Abbruzzese G, Berardelli A (2004). Central effects of botulinum toxin type A: evidence and supposition. Mov. Disord.

[b13] Delwaide PJ, Oliver E (1988). Short-latency autogenic inhibition (IB inhibition) in human spasticity. J. Neurol. Neurosurg. Psychiatry.

[b14] Filippi GM, Errico P, Santarelli R, Bagolini B, Manni E (1993). Botulinum A toxin effects on rat jaw muscle spindles. Acta Otolaryngol. (Stockh).

[b15] Floeter MK, Andermann F, Andermann E, Nigro M, Hallett M (1996). Physiological studies of spinal inhibitory pathways in patients with hereditary hyperekplexia. Neurology.

[b16] Girlanda P, Quartarone A, Sinicropi S, Nicolosi C, Roberto ML, Picciolo G (1997). Botulinum toxin in upper limb spasticity: study of reciprocal inhibition between forearm muscles. NeuroReport.

[b17] Gracies J-M, Lugassy M, Weisz DJ, Vecchio M, Flanagan S, Simpson DM (2009). Botulinum toxin dilution and endplate targeting in spasticity: a double-blind controlled study. Arch. Phys. Med. Rehabil.

[b18] Hagenah R, Benecke R, Wiegand H (1977). Effects of type A botulinum toxin on the cholinergic transmission at spinal Renshaw cells and on the inhibitory action at Ia inhibitory interneurones. Naunyn Schmiedebergs Arch. Pharmacol.

[b19] Hesse S, Krajnik J, Luecke D, Jahnke MT, Gregoric M, Mauritz KH (1996). Ankle muscle activity before and after botulinum toxin therapy for lower limb extensor spasticity in chronic hemiparetic patients. Stroke.

[b20] Hidler JM, Carroll M, Federovich EH (2007). Strength and coordination in the paretic leg of individuals following acute stroke. IEEE Trans. Neural Syst. Rehabil. Eng.

[b21] Hopkins WG (1984). Axon growth in tendon organs of botulinum-paralyzed neonatal mouse muscles. Exp. Neurol.

[b22] Hutton RS, Roy RR, Edgerton VR (1988). Coexistent Hoffmann reflexes in human leg muscles are commonly due to volume conduction. Exp. Neurol.

[b23] Iles JF, Baderin R, Tanner R, Simon A (2007). Human standing and walking: comparison of the effects of stimulation of the vestibular system. Exp. Brain Res.

[b24] Kaji R (2013). Direct central action of intramuscularly injected botulinum toxin: is it harmful or beneficial?. J. Physiol.

[b25] Kaji R, Osako Y, Suyama K, Maeda T, Uechi Y, Iwasaki M (2010). Botulinum toxin type A in post-stroke lower limb spasticity: a multicenter, double-blind, placebo-controlled trial. J. Neurol.

[b26] Knutsson E, Mårtensson A (1980). Dynamic motor capacity in spastic paresis and its relation to prime mover dysfunction, spastic reflexes and antagonist co-activation. Scand. J. Rehabil. Med.

[b27] Knutsson E, Richards C (1979). Different types of disturbed motor control in gait of hemiparetic patients. Brain.

[b28] Manni E, Bagolini B, Pettorossi VE, Errico P (1989). Effect of botulinum toxin on extraocular muscle proprioception. Doc. Ophthalmol.

[b29] Marchand-Pauvert V, Nielsen JB (2002). Modulation of heteronymous reflexes from ankle dorsiflexors to hamstring muscles during human walking. Exp. Brain Res.

[b30] Marchand-Pauvert V, Aymard C, Giboin L-S, Dominici F, Rossi A, Mazzocchio R (2013). Beyond muscular effects: depression of spinal recurrent inhibition after botulinum neurotoxin A. J. Physiol.

[b31] Mazzocchio R, Spidalieri R, Dominici F, Popa T, Hallett M, Rossi A (2007). Putative central effects of botulinum toxin, possibly mediated by changes in Renshaw cell activity, following intramuscular injection in humans. Mov. Disord.

[b32] Misiaszek JE, Stephens MJ, Yang JF, Pearson KG (2000). Early corrective reactions of the leg to perturbations at the torso during walking in humans. Exp. Brain Res.

[b33] Modugno N, Priori A, Berardelli A, Vacca L, Mercuri B, Manfredi M (1998). Botulinum toxin restores presynaptic inhibition of group Ia afferents in patients with essential tremor. Muscle Nerve.

[b34] Neckel N, Pelliccio M, Nichols D, Hidler J (2006). Quantification of functional weakness and abnormal synergy patterns in the lower limb of individuals with chronic stroke. J. Neuroeng. Rehabil.

[b35] Newham DJ, Hsiao SF (2001). Knee muscle isometric strength, voluntary activation and antagonist co-contraction in the first six months after stroke. Disabil. Rehabil.

[b36] Nielsen J, Kagamihara Y (1992). The regulation of disynaptic reciprocal Ia inhibition during co-contraction of antagonistic muscles in man. J. Physiol.

[b37] Okuma Y, Lee RG (1996). Reciprocal inhibition in hemiplegia: correlation with clinical features and recovery. Can. J. Neurol. Sci.

[b38] On AY, Kirazli Y, Kismali B, Aksit R (1999). Mechanisms of action of phenol block and botulinus toxin Type A in relieving spasticity: electrophysiologic investigation and follow-up. Am. J. Phys. Med. Rehabil.

[b39] Panizza M, Castagna M, Saibene A, di Summa L, Grioni G, Nilsson J (2000). Functional and clinical changes in upper limb spastic patients treated with botulinum toxin (BTX). Funct. Neurol.

[b40] Perry J, Waters RL, Perrin T (1978). Electromyographic analysis of equinovarus following stroke. Clin. Orthop. Relat. Res.

[b41] Petersen N, Morita H, Nielsen J (1999). Modulation of reciprocal inhibition between ankle extensors and flexors during walking in man. J. Physiol.

[b42] Pierrot-Deseilligny E, Burke D (2012). Spinal and corticospinal mechanisms of movement.

[b43] Pierrot-Deseilligny E, Morin C, Bergego C, Tankov N (1981). Pattern of group I fibre projections from ankle flexor and extensor muscles in man. Exp. Brain Res.

[b44] Priori A, Berardelli A, Mercuri B, Manfredi M (1995). Physiological effects produced by botulinum toxin: changes in reciprocal inhibition between forearm muscles. Brain.

[b45] Rosales RL, Dressler D (2010). On muscle spindles, dystonia and botulinum toxin. Eur. J. Neurol.

[b46] Rosales RL, Arimura K, Takenaga S, Osame M (1996). Extrafusal and intrafusal muscle effects in experimental botulinum toxin-A injection. Muscle Nerve.

[b47] Stephens MJ, Yang JF (1996). Short latency, non-reciprocal group I inhibition is reduced during the stance phase of walking in humans. Brain Res.

[b48] Torii Y, Akaike N, Harakawa T, Kato K, Sugimoto N, Goto Y (2011). Type A1 but not type A2 botulinum toxin decreases the grip strength of the contralateral foreleg through axonal transport from the toxin-treated foreleg of rats. J. Pharmacol. Sci.

[b49] Vinti M, Costantino F, Bayle N, Simpson DM, Weisz DJ, Gracies J-M (2012). Spastic cocontraction in hemiparesis: effects of botulinum toxin. Muscle Nerve.

[b50] Wargon I, Lamy JC, Baret M, Ghanim Z, Aymard C, Pénicaud A (2006). The disynaptic group I inhibition between wrist flexor and extensor muscles revisited in humans. Exp. Brain Res.

[b51] Weerdesteyn V, Geurts M, de Niet HJR, van Duijnhoven ACH (2008). Falls in individuals with stroke. J. Rehabil. Res. Dev.

[b52] Wiegand H, Wellhöner HH (1977). The action of botulinum A neurotoxin on the inhibition by antidromic stimulation of the lumbar monosynaptic reflex. Naunyn Schmiedebergs Arch. Pharmacol.

[b53] Yanagisawa N, Tanaka R, Ito Z (1976). Reciprocal Ia inhibition in spastic hemiplegia of man. Brain.

